# Fast anther dehiscence status recognition system established by deep learning to screen heat tolerant cotton

**DOI:** 10.1186/s13007-022-00884-0

**Published:** 2022-04-21

**Authors:** Zhihao Tan, Jiawei Shi, Rongjie Lv, Qingyuan Li, Jing Yang, Yizan Ma, Yanlong Li, Yuanlong Wu, Rui Zhang, Huanhuan Ma, Yawei Li, Li Zhu, Longfu Zhu, Xianlong Zhang, Jie Kong, Wanneng Yang, Ling Min

**Affiliations:** 1 National Key Laboratory of Crop Genetic Improvement, Huazhong Agricultural University, Wuhan, 430070 Hubei China; 2 Forestry and Fruit Tree Research Institute, Wuhan Academy of Agricultural Sciences, Wuhan, 430075 China; 3 Institute of Economic Crops, Xinjiang Academy of Agricultural Sciences, Xinjiang, 830091 China

**Keywords:** Cotton anther, Deep learning, Faster R-CNN, YOLOv5, Model ensemble, High temperature stress

## Abstract

**Background:**

From an economic perspective, cotton is one of the most important crops in the world. The fertility of male reproductive organs is a key determinant of cotton yield. Anther dehiscence or indehiscence directly determines the probability of fertilization in cotton. Thus, rapid and accurate identification of cotton anther dehiscence status is important for judging anther growth status and promoting genetic breeding research. The development of computer vision technology and the advent of big data have prompted the application of deep learning techniques to agricultural phenotype research. Therefore, two deep learning models (Faster R-CNN and YOLOv5) were proposed to detect the number and dehiscence status of anthers.

**Result:**

The single-stage model based on YOLOv5 has higher recognition speed and the ability to deploy to the mobile end. Breeding researchers can apply this model to terminals to achieve a more intuitive understanding of cotton anther dehiscence status. Moreover, three improvement strategies are proposed for the Faster R-CNN model, where the improved model has higher detection accuracy than the YOLOv5 model. We have made three improvements to the Faster R-CNN model and after the ensemble of the three models and original Faster R-CNN model, R^2^ of “open” reaches to 0.8765, R^2^ of “close” reaches to 0.8539, R^2^ of “all” reaches to 0.8481, higher than the prediction results of either model alone, which are completely able to replace the manual counting results. We can use this model to quickly extract the dehiscence rate of cotton anthers under high temperature (HT) conditions. In addition, the percentage of dehiscent anthers of 30 randomly selected cotton varieties were observed from the cotton population under normal conditions and HT conditions through the ensemble of the Faster R-CNN model and manual counting. The results show that HT decreased the percentage of dehiscent anthers in different cotton lines, consistent with the manual method.

**Conclusions:**

Deep learning technology have been applied to cotton anther dehiscence status recognition instead of manual methods for the first time to quickly screen HT–tolerant cotton varieties. Deep learning can help to explore the key genetic improvement genes in the future, promoting cotton breeding and improvement.

**Supplementary Information:**

The online version contains supplementary material available at 10.1186/s13007-022-00884-0.

## Background

Cotton is an economically important crop, and its reproductive development is susceptible to a variety of adverse stresses that affect its yield and quality. The reproductive organs of cotton include stamens and pistils, and stamens are more sensitive to heat stress than female organs [19]. In many summer crops, reproductive organ abortion caused by high temperatures (HT) is manifested by normal development of the female reproductive system and abnormal development of the male reproductive system, causing failure to produce functional pollen or deficiency of the anthers to achieve dehiscence properly to release pollen. Anther development is a complex processing, going from sporogenic cells to anther dehiscence, and it has been divided into 14 periods by studying a variety of male sterile mutants [25]. Anther dehiscence, the final step in anther development, includes three processes: secondary thickening of the inner wall of the anther chamber, degradation of the septum cells, and dehiscence of the cleft which ultimately allow the release of pollen [10]. Therefore, anther dehiscence is directly related to the probability of fertilization in cotton. If we can obtain phenotypic data on anther dehiscence quickly and accurately to conduct genome-wide association analysis, then we can easily obtain the functional genes related to anther dehiscence. It is also important to analyze the molecular mechanism of cotton male reproductive organs in response to stress.

In the past, the acquisition of cotton dehiscent or indehiscent anther number data from pictures relied mainly on visual observation and manual counting. It is difficult to guarantee the accuracy of visual readings because anther growth is intermingled, resulting in an unclear definition of individual anthers; in addition, the background and foreground of anthers are easily confused. Moreover, a larger amount of anther data is needed to judge the anther growth and dehiscence status of individual plants in populations under different conditions. However, it is obviously difficult to achieve this accurately and quickly with manual methods.

After 2012, the concept of deep learning was proposed. Deep learning techniques have evolved rapidly in the past few years. The YOLO series, Faster-RCNN and single shot multibox detector (SSD) are three important deep learning neural network models [13]. Faster-RCNN mainly extracts preselected boxes and then performs deep learning classification. The image detection process of Faster-RCNN includes region proposal extraction, candidate feature frame extraction, and candidate feature frame classification. The YOLO model cleverly uses the idea of regression by taking the whole image as input, dividing it into several boxed regions, removing individual boxes with very low relevance by setting specific thresholds, and finally selecting the highest scoring region with a nonmaximum suppression algorithm. Through classification and extraction of image features and end-to-end training of deep learning models, computers can accurately detect specific content in images. By building different datasets and replacing deep learning network architectures, researchers can obtain network models that are more suitable for research purposes than previous approaches.

The application of target detection technology to agriculture using machine learning has been very extensive [1, 5, 8, 26]. In maize, a parabolic model has been used to mine the diversity of stem-end meristematic tissues and to find candidate genes that correlate with the transport of phytohormones, cell division, and cell size by GWAS [29]. In rice, the ratio of spikes to leaves, a new trait of rice, has been extracted using a feature pyramid network mask model that has achieved leaf and spike recognition accuracies of 0.98 and 0.99, respectively [30]. Ferentinos has designed a convolutional neural network model to solve the problem of early plant disease detection. Through the deep learning method, several model structures have been trained with plant leaf images and have identified the corresponding plant leaf lesions with 99.53% accuracy. The model has become a powerful tool for the early diagnosis and early warning of plant leaf diseases and can be further improved. Therefore, the system can be used in real time in a real cultivation environment [4]. Ubbens et al. have designed an open-source deep learning tool called Deep Plant Phenomics for plant phenotypic deep learning. This tool provides pretrained neural networks for several common plant phenotypic tasks including leaf counting, image classification and age regression. Botanists can use the provided neural networks trained by this platform to train their plant phenotypes [26]. Genze et al. have proposed a convolutional neural network-based seed germination status recognition system that can automatically identify seed categories (including maize, rye, and pearl millet) in petri dishes to and automatically determine whether the seeds are germinating. The system achieves an average accuracy of 94% on test data and can help seed researchers to better determine seed quality and performance [6]. Scientists use hyperspectral imaging technology to collect spectral and image information from maize seeds and combine convolutional neural networks and support vector machines to model and train spectral datasets and image datasets. Such models can quickly detect the vigor state of seeds and simultaneously predict their germination status, providing a framework to advance research on seed germination [17, 18]. A MobileNetv2-YOLOv3-based model that combines pretraining methods such as hybrid training and migration learning to improve the generalization of the model for the early identification of tomato leaf spot disease has been proposed [12]. Image processing and machine learning techniques have been used to accurately classify the three stages of plant growth and soil type for different germplasms of two species of red clover and alfalfa. The accuracy on test data was shown to be more than 90% [24]. The researchers developed a cotton florescence detection system based on Faster R-CNN, which is installed on the ground mobile system (GPhenoVision), which can detect and calculate new flowers on a given date, and monitor cotton flowering growth and yield prediction on the field [9]. To achieve the classification of cotton leaf spots by small sample learning, a metric-based learning method was developed to extract cotton leaf spot features and classify sick leaves [11]. However, no reports of machine learning-based anther identification systems in academia, which motivated us to build a deep learning-based anther identification system for cotton.

In this study, using YOLOv5 [18, 20–22, 28] and Faster R-CNN [23], combined with a variety of data augmentation methods, a cotton anther recognition model based on deep learning is obtained. This model can quickly recognize batch input cotton anther images, detect dehiscent and indehiscent anthers, and obtain phenotypic data. Using this model to detect 30 randomly selected cotton varieties, it is found that high temperature (HT) could significantly reduce the anther dehiscence rate, which can be used as a basis for screening HT tolerant germplasms and help to locate HT tolerant genes.

## Materials and methods

### Material growing and dataset acquisition

In total, 510 cotton lines from natural populations were planted in 2016–2019 in experimental cotton fields at Huazhong Agricultural University, Wuhan, Hubei (113.41 E, 29.58 N), Turpan, Xinjiang (89.19 E, 42.91 N), and Alar, Xinjiang (81.29 E, 40.54 N). At Wuhan, the field was planted at a density of 27,000 plants per hectare with each row including more than 12 individuals. At Alar and Turpan, Xinjiang, the fields were set up with two streets and planted at a density of 195,000 plants per hectare. More than 30 individuals of each line were arranged in rows. Cotton anther images were collected each year at each location three days after the onset of normal temperatures and after high temperatures during bloom.

**Fig.1:**
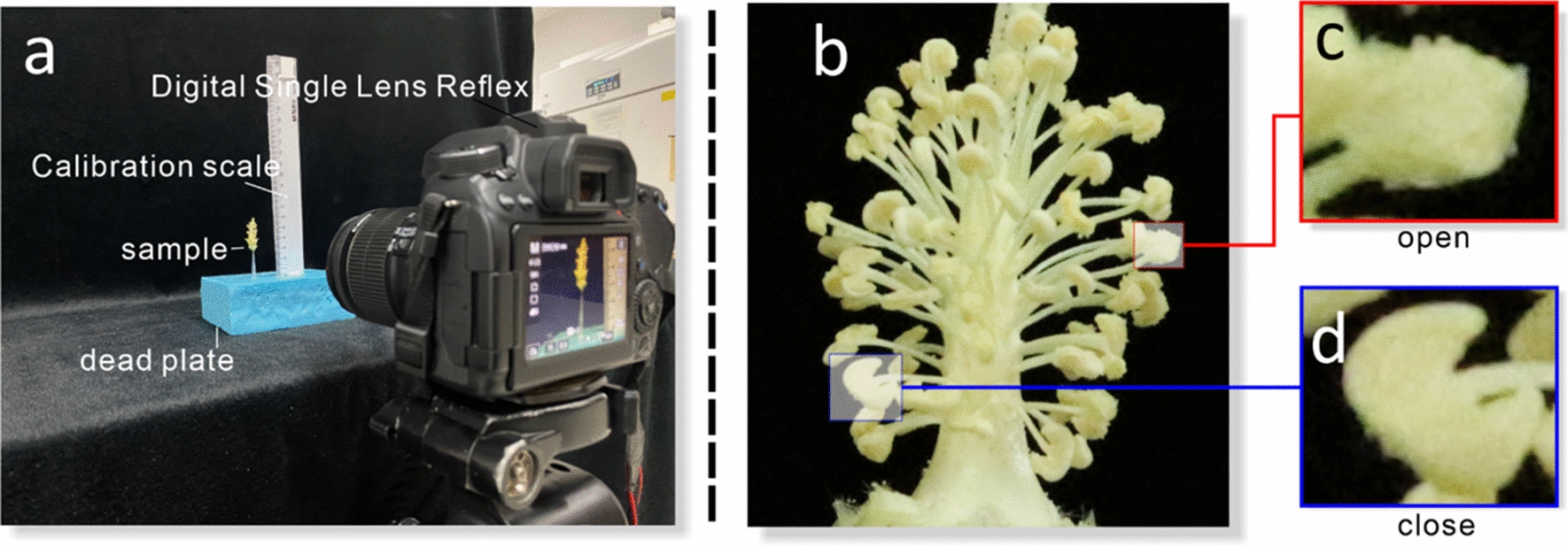
Data acquisition. **a** The image dataset captures the platform scene. **b** Image of cotton anthers. **c** The surface of dehiscent cotton anther (open) is rough in the image. **d** The surface of an indehiscent cotton anther (close) is smooth in the image

A Canon 70 d HD digital camera was used throughout the acquisition of a research image dataset. To prevent the negative interference of background with the subsequent machine recognition effort, a black curtain was used as the photo background for the experiments. In the actual image collection process, it was found that the cotton anthers were surrounded by cotton petals, and the anthers growing at the root of the style were not easily captured by the camera, therefore, taking the pictures directly was not conducive to the accurate collection of data. Thus, it was necessary to preprocess the cotton flowers before acquiring the pictures by stripping the cotton petals and fixing the anther sides. To prevent overfitting and to overcome issues related to insufficient training data, the same anthers were included in multiple distant near-field images (Fig. 1). Finally, a total of 38,895 high-definition RGB whole anther images were acquired.

**Fig. 2:**
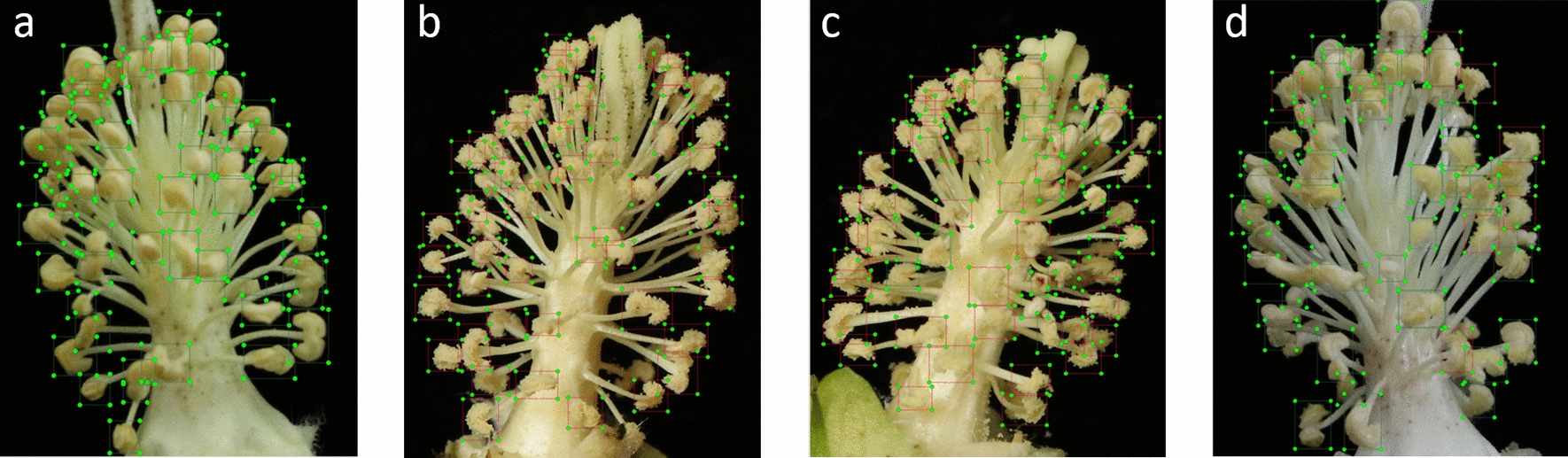
Image labeling. The above figures are manually marked cotton anther images using the “Labelimg" software. Green boxes represent indehiscent anthers and red boxes represent dehiscent anthers. When the image labeling was finished, the corresponding location information of the image was saved in VOC format along with the name of the image **a** All the anthers are indehiscent. **b** All the anthers are dehiscent. **c** Dehiscent anthers account for the majority. **d** Indehiscent anthers account for the majority

Morphologically, dehiscent anthers are rough and grainy because the released pollen adheres to anther edges, while indehiscent anthers have smooth edges, because no pollen is released. Therefore, the obtained cotton anther images were annotated using “Labelimg” image annotation software, as shown in Fig. 2. The image boundary of each visible cotton anther is captured within an annotation box that reduces the influence of background on model training, and contains a labele, “open” or “close” to distinguish dehiscent and indehiscent anthers, respectively. A total of 2845 images were annotated one by one. The images were used as the input dataset and were randomly divided into a training set and validation set with a ratio of 7:3 (Additional file 1: Table S1).

### Experimental operation environment

The hardware environment used in this study shown in Additional file 2: Table S2. The training environment is Python, Open-cv, Cuda, whereas the frameworks used in this study are Paddle and Pytorch.

## Results

### YOLOv5 model design

YOLOv5 is a typical one-stage detection model, which increases the detection speed by 50% compared with the previous generation YOLOv4, with a model size only 1/10 of that of YOLOv4. The adaptive anchor frame calculation and the use of a focus structure enhance the accuracy of the model for small target recognition. At the same time, the model has four network models with different depths, allowing for the best balance between detection accuracy and recognition speed to be found. It is very common for cotton anthers to block each other in the image; hence, the obscured anthers are easily ignored in the final output of the prediction box. To screen the prediction box, usually used NMS or soft-NMS algorithm is used. The idea of the NMS algorithm is as follows. For a certain category X, having N candidate boxes, the candidate boxes are sorted by their confidence, and the highest confidence Box A is selected. The other candidate Boxes Bi (i = 1, 2, 3…) are compared with the highest confidence Box A, and an IoU threshold is set. If its IoU is higher than this threshold, the candidate Box B1 is discarded. Then the candidate Box B2’s IoU is compared with that of the highest confidence Box A. After several iterations, only prediction boxes that have an IoU lower than the set IoU value are retained. Although this method can prevent the same target from being repeatedly selected by multiple prediction boxes, it cannot prevent overlapping or occluded targets from being ignored.

The idea of Soft-NMS is that M is the current highest scoring box and Bi is the pending box. The larger the IoU of Bi and M, the greater the reduction in score Si of Bi drops, preventing the score to go directly to zero as in NMS. This method can effectively retain anther images that overlap and ensure the accuracy of the identification results. The linear weighting formula for Soft-NMS can be expressed as:

$$ S_{i} \, = \,\left\{ \begin{gathered}   S_{i}  \hfill \\   S_{i} \left( {1 - IOU\left( {M,b_{i} } \right)} \right) \hfill \\  \end{gathered}  \right.\;\begin{array}{*{20}c}    {IOU\left( {M,b_{i} } \right) \le N_{t} }  \\    {IOU\left( {M,b_{i} } \right) \ge N_{t} }  \\   \end{array} $$

Thus, when the prediction box is screened while, using the NMS algorithm, the anther images with the highest confidence are exclusively retained. Therefore, we used YOLOv5 with the soft-NMS algorithm [2] to screen the prediction box.

### Faster R-CNN model design

Faster R-CNN is a classical two-stage object detection network. The network model structure is mainly composed of four parts: feature extraction, region proposal, classification, and roi pooling. The comprehensive performance of this network has been greatly improved, especially for the detection accuracy of small targets. The cotton anther belongs to the range of small targets to be detected in the whole image, so we trained the Faster R-CNN model to identify the anther dehiscence state with a better detection effect.

Conv layers are usually used to extract the feature maps of the input image, through a classical CNN network target detection method, that mainly includes three layers of conv, pooling, and RelU. The extracted feature maps will be called by subsequent region proposal networks and classification networks. The convlayers structure, contains 13 conv layers, 13 RelU layers, and 4 pooling layers. The Faster R-CNN has an ingenious detail in the convlayers; it performs augmentation treatment on all convolutional layers, and fills a layer in the outer layer of the input matrix, so that the matrix is larger than before, and the images that have been treated in this way are deconvoluted again. After the convolution operation, the image is kept consistent with the size of the input image. The matrix size is unchanged when the image goes through the conv layer and RelU layer, and will change to 1/2 of the original size after going through the pooling layer, so that when going through the conv layers structure, the size of the input matrix changes to 1/16 of the original size; thus, the resulting feature maps can all correspond one-to-one with the original graph.

Conventional detection methods usually use a sliding window or the selective search method to acquire detection frames, whereas Faster R-CNN discards traditional methods and directly generates detection frames using region proposal networks, which greatly enhances the detection frame generation speed. The region proposal network structure is actually divided into two processes: the first process uses softmax classification anchors to obtain the foreground and background (the detection target is the foreground), and the second process calculates the bounding box regression offset for anchors to obtain the exact proposal. Finally, the proposal layer is responsible for integrating foreground anchors and bounding box regression offset to obtain proposals, while simultaneously removing proposals too small beyond the boundary. The entire Faster R-CNN network arrives at the proposal layer, completing detection targets, and the next two structures are mainly used for image recognition.

For the traditional CNN network, the input image of the model must be a fixed size, and the output of the model must be a fixed vector or matrix. In practical applications, there are two solutions for images of different sizes: cut the picture to a fixed size or warp the image to a fixed size. However, these solutions will either cause the loss of image information, or lead to changes in the shape information of the image. Therefore, structure roi pooling is proposed in Faster R-CNN to solve the problem of different image sizes. Roi pooling is mainly responsible for collecting feature maps and proposal boxes, calculating proposal feature maps, and sending them to the subsequent identification layer. First, the proposal is mapped to the same scale as the feature maps, and then the vertical and horizontal directions of each proposal are divided into seven parts, so that the output of different proposal sizes is 7*7, realizing a fixed-length output.

To classify using the obtained proposal feature maps, the structure calculates which category each proposal belongs to through full connection layers and softmax, and outputs the probability vector. At the same time, the position offset of each proposal is obtained again by bounding box regression, which is used to return a more accurate target detection box.

The loss function of the object detection network of Faster R-CNN is shown in the formula below:

$$ L_{reg} \left( {t_{i} ,t_{i}^{*} } \right) = \sum\limits_{{i \in \left\{ {x,y,w,h} \right\}}} {smooth_{L1} \left( {t_{i} - t_{i}^{*} } \right)} $$

$$ soomth_{L1} (x) = \left\{ {\begin{array}{ll} {\begin{array}{*{20}c} {0.5x^{2} } & {if} \\ \end{array} \left| x \right| < 1} \\ {\begin{array}{*{20}c} {\left| x \right| - 5} & {otherwise} \\ \end{array} } \\ \end{array} } \right. $$

In the above mentioned formula, i represents the anchors index; t represents the predicted bounding box; t* represents the true ground box corresponding to the positive anchor; and (x,y), w and h represents the center point coordinates of the box, width, and height, respectively.

### Data augmentation

In deep learning, in general, the greater the number of samples, the better the effect of the trained model. However, in the actual situation, due to different lighting, shooting angle conditions, as well as the state of the sample itself, we are often unable to collect all of the possibilities for the sample, necessitating data augmentation of the sample and artificial creation of more samples. Increasing the amount of training data can improve the generalization ability of the model, while increasing- the amount of noise data can improve the robustness of the model. In addition, more data can make the model less prone to overfitting in the training process. Therefore, we have tried several data augmentation methods for the cotton anther dataset, hoping to obtain a more suitable model for this study through the enhanced dataset.

### Auto augment

This approach creates a search space for data-enhanced policies in which a policy contains many subpolicies and randomly selects one subpolicy for each image in a small batch dataset. Each sub strategy consists of two operations, that consists of an image processing function similar to traction, rotation, or shearing, and the probability and magnitude of applying those functions, using a search algorithm directly on the dataset to find the best data augmentation strategy. 

### Random resize

Random Resize scales the new image to the same pixel size as the original image by randomly clipping the original image in the dataset according to the random aspect ratio.

### Random flip

Random flip is a common method of data augmentation, that generates new dataset samples by randomly flipping the original image of the dataset up and down or left and right.

### Mixup

Mixup is a data augmentation method for mixing two samples and label data at their corresponding ratios and then generating a new sample and label data. Suppose x_1_ is a sample of batch one, y_1_ is the label corresponding to the sample of batch one; x_2_ is the sample of batch two,${\mathrm{y}}_{2}$ y_2_ is the sample corresponding label of batch two, and x_mix_ and y_mix_ are the newly generated sample and corresponding label, respectively. λ is the mixing coefficient resulting from the hyperparametric α and β conducted beta distributions. The principal formula of the mixup method can be expressed as:

$$ x_{mix} = \lambda x_{1} + (1 - \lambda )x_{2} $$

$$ y_{mix} = \lambda y_{1} + (1 - \lambda )y_{2} $$

$$ \lambda \sim Beta(\alpha ,\beta )\,\quad \alpha ,\beta \in \left[ {0, + \infty } \right] $$

According to the study, we know that as the hyperparameters α and β increase, the error and generalization ability of the network training will increase. When the beta distribution of the mixing coefficient λ is α = β = 0, the network reverts to the ERM (empirical risk minimization) principle to minimize the training data average error; the beta distribution of the mixing coefficient λ has the best generalization ability and robustness. This method can make full use of all the pixel information, but at the same time also introduces some unnecessary pseudopixel information.

### Cutmix

Cutmix [31] cuts some regions in the sample, randomly fills in the pixel values of other samples in the dataset, and distributes the final classification results according to a certain proportion. Compared with mixup, cutmix can prevent the occurrence of nonpixel information in the training process. Filling the pixel information of other regions with the missing area of cut can further enhance the positioning ability of the model. At the same time, this method will not increase the training and reasoning burden of the model.

### GridMask

By generating a mask with the same resolution as the original image, GridMask multiplies the mask with the original image to obtain a new image. The pixel value of the new image in the fixed area is 0, which is essentially a regularization method. Compared with directly changing the network structure, GridMask only needs to be augmented when the image is input.

### Normalized

We usually use this method after data augmentation. Normalizing the pixel value of the image and scaling the pixel value to [0, 1] can prevent the attributes of the large value interval from excessively dominating the attributes of the decimal value interval, and at the same time avoid numerical complexity in the calculation process.

**Fig. 3:**
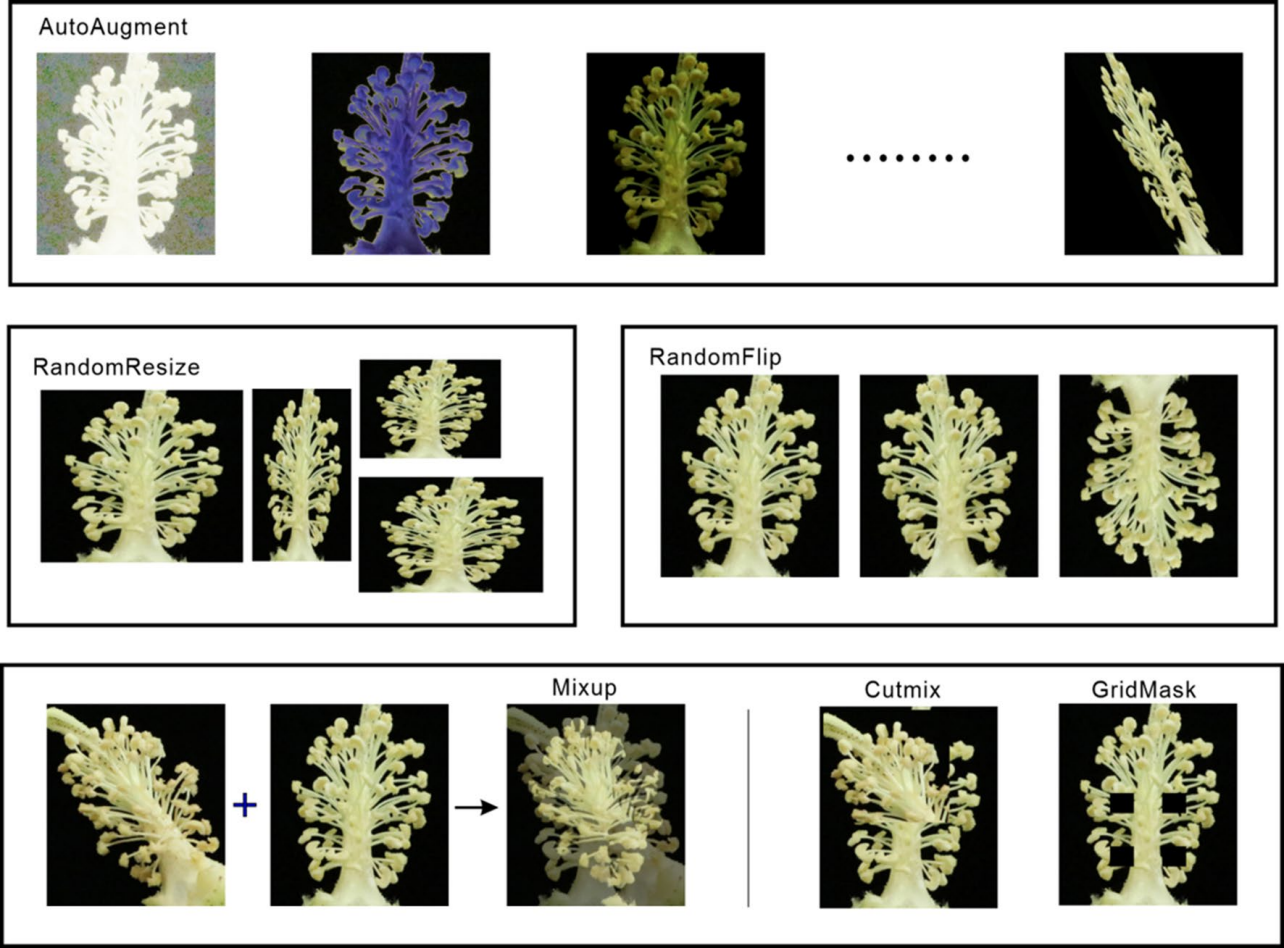
Data augmentation. The above images show the effect of different data augmentation methods on the same cotton anther image

The data augmentation process of this study is shown in the Fig. 3.

### Model training

**Fig. 4:**
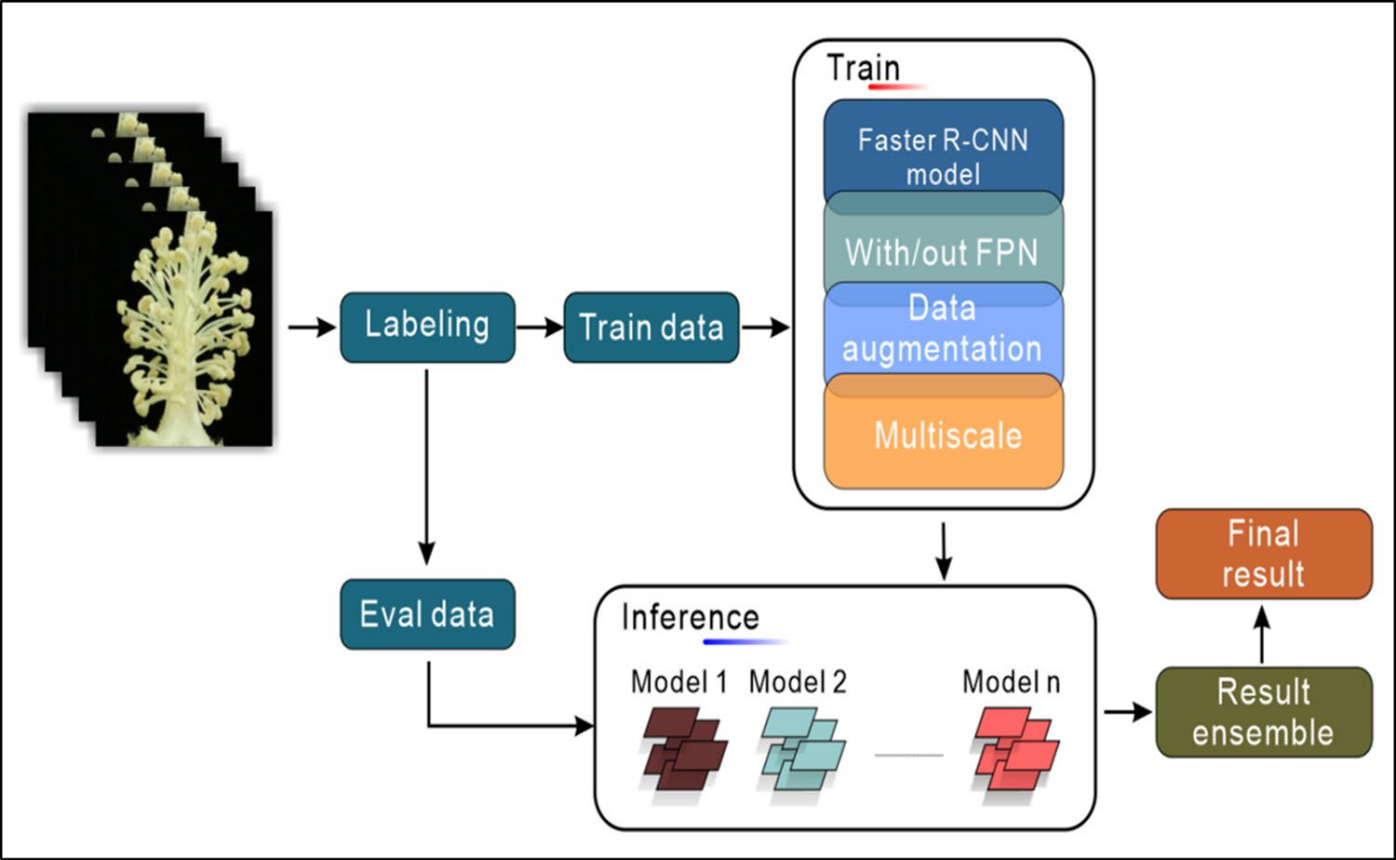
Model ensembles. Integrated flow chart of cotton anther recognition model ensembles

In this study, comparative experiments and control variables between the YOLOv5 and Faster-RCNN models were used, and various data demonstration methods, such as mixing and mixed cutting were generated to train for sample imbalance, and to verify the performance of different models and training methods on the same evaluation index of the validation set. First, the homemade dataset was segmented and analyzed, and VOC format was used to store the training, test and verification sets. Second, the model was trained by considering whether the data demonstration algorithm was added or not. Finally, the cosine strategy was used to periodically attenuate the learning rate. The training stopped when the average loss remained stable. The training process of the Faster R-CNN model of this study is shown in Fig. 4.

**Fig. 5:**
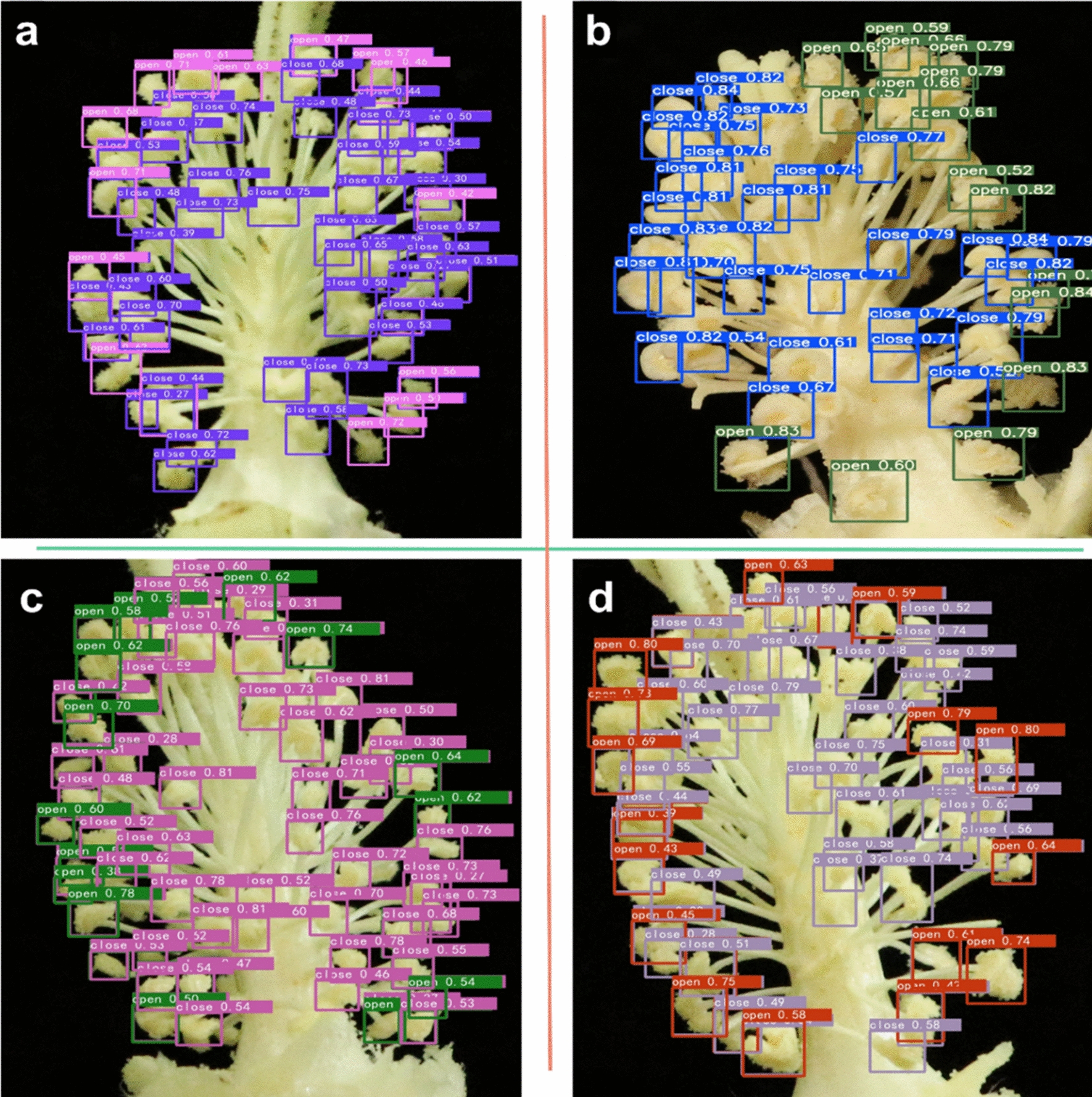
Cotton anther identification effect graph.** a** The purple box marks an indehiscent cotton anther, and the pink box marks a dehiscent cotton anther. **b** The blue box marks an indehiscent cotton anther, and the gray box marks a dehiscent cotton anther. **c** The pink box marks an indehiscent cotton anther, and the green box marks a dehiscent cotton anther. **d** The gray box marks an indehiscent cotton anther, and the red box marks a dehiscent cotton anther. In each test, the colors of the prediction boxes with different labels were randomly generated

The models obtained by different training strategies were tested on the test set, and the prediction results of multiple models were obtained. The results of the four groups of comparison experiments indicated that the proposed Faster R-CNN neural network with data augmentation and FPN (feature pyramid networks) structure on Multi-Scale [3] could effectively detect dehiscence and indehiscence in cotton anther images. Compared with other methods, this method has significant advantages in recognition accuracy. The recognition effect is shown in Fig. 5. The final result was obtained by the prediction results of ensembles of multiple models.

### Model comparison

#### Metrics to evaluate the proposed method

In this study, we used mAP@0.5:0.95, as well as MAD (mean absolute deviation) and R^2^ as the evaluation indicators of the model. The indicators are explained as follows:

mAP@0.5:0.95 is the process of increasing intersection over union (IoU) from 0.5 to 0.95 with steps of 0.05. The mAP corresponding to each IoU is added to obtain the average value of mAP in this process. The formula is expressed as follows:

$$ P = \frac{{T_{P} }}{{P_{N} }} $$

$$ R = \frac{{T_{P} }}{{T_{N} }} $$

$$ AP = \int\limits_{0}^{1} {P(R)dR} $$

In the above formula, T_P_ is the correct number of categories identified by the model, P_N_ is the total number of categories identified by the model, and T_N_ ${T}_{N}$ is the true number of categories. Averaging the AP values of all categories is called mAP.

We took the absolute value of the absolute error between the measured value and the real value and then calculated the average value, calling it MAD. Because the deviation is an absolute value, the positive and negative values will not be offset; thus, the mean absolute error can reflect the actual situation of the predicted value deviation. The smaller the value is, the closer the prediction of the model is to reality.

The main purpose of this study was to develop a deep learning model that can quickly and accurately identify anther dehiscence and explore the influence of high temperature stress on cotton anther dehiscence. In the model identification phase, we identify the location of the cotton anther without strict requirements, and a model was needed to recognize the anther number by artificial observations. Then this number was used as an accurate value for the validation set, which uses the correlation coefficient between predicted values and the accurate value as the main evaluation index of the model.

To facilitate the follow-up description, the dehiscent anther is referred to as ‘open’, the non-dehiscent anther is referred to as ‘close’, and all cotton anthers are abbreviated as ‘all’.

### Comparison of detection results of Faster R-CNN and YOLOv5

**Fig. 6:**
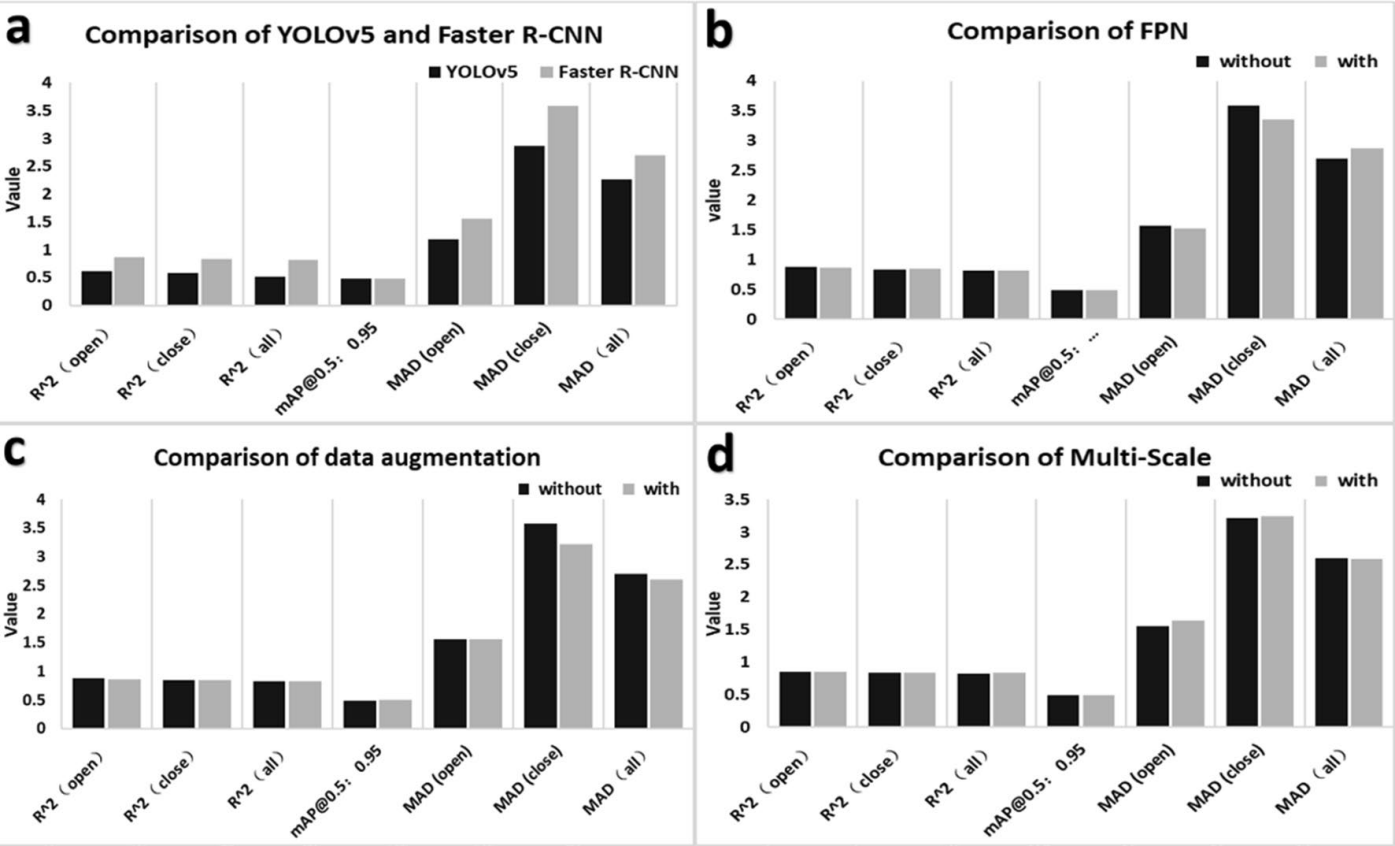
Comparison of different models.** a** Comparison of YOLOv5 and Faster R-CNN. The YOLOv5 model has a higher recognition speed than Faster R-CNN, and the Faster R-CNN model has a higher detection accuracy than YOLOv5. **b** Comparison of with or without FPN (Feature Pyramid Networks) The mAP@0.5:0.95 of the improved model increased by 0.002, R^2^ ${R}^{2}$ of "close" class increased by 0.003, and R^2^ of the "open" class and "all" the decreased slightly. **c** Comparison of with or without data augmentation. The improved model has a slight decline in the number of R^2^ in the open category and an improvement in other evaluation indicators. **d** Comparison of with or without data Multi-Scale. The results showed that the mAP@0.5:0.95 of the model was improved by 0.003 after Multi-Scale training. ${R}^{2}$ R^2^ in the "open" and "close" categories fell by 0.0092 and 0.0007, respectively. R^2^ ${R}^{2}$ in the "all" category increased to 0.0086. "open" and "close" represent dehiscent and indehiscent anthers, respectively

Faster R-CNN and YOLOv5 are used to train the same training set, the test results are compared on the same test set, and a correlation between the test results and the accurate numbers of manual labeling is performed. YOLOv5 using Darknet53 as the backbone network is a typical single-stage model, while Faster R-CNN using Res101 as the backbone network is a standard two-stage model. Obviously, YOLOv5 is more advantageous in detection speed. A comparison of the two models is shown in Fig. 6a. Through training and validation, we found that the mAP@0.5:0.95 of YOLOv5 was 0.485, while the mAP@0.5:0.95 of Faster R-CNN was 0.478. In mAP@0.5:0.95, YOLOv5 was 0.007 higher than Faster R-CNN. In terms of the evaluation index of R^2^ in the validation set, Faster R-CNN was 0.8712 in the category of "open" and 0.8373 in the category of "close", and 0.82 in the category of "all", which were 0.2523, 0.2619, and 0.3104 higher than YOLOv5, respectively. This may be due to the interference of location information. Although YOLOv5 has a slightly higher mAP@0.5:0.95, R^2^ is far lower than Faster R-CNN (Additional file 3: Table S3). Since quantitative accuracy is our primary research goal, we decided to further optimize the two-stage Faster R-CNN model.

### Comparison of detection results with or without FPN

**Fig. 7:**
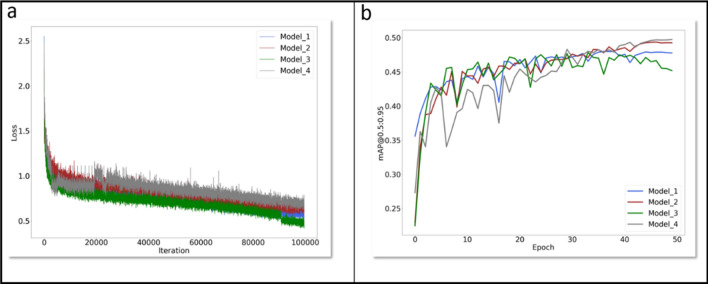
mAP@0.5:0.95 curves and LOSS curves. **a** mAP@0.5:0.95 curves. **b** LOSS curves. *Model 1* is the Faster R-CNN with FPN structure. *Model 2* is the Faster R-CNN with data augmentation and FPN structure. *Model 3* is the traditional Faster R-CNN. *Model 4* is the Faster R-CNN with Multi-Scale data augmentation and FPN structure. Epoch: All the data were sent into the network to complete a process of forward calculation and backpropagation. mAP@0.5:0.95 is the process of increasing IoU from 0.5 to 0.95 according to the span of 0.05. The mAP corresponding to each IoU was added to obtain the average value of mAP in this process

To further improve the detection effect of the Faster R-CNN model, the FPN structure was added into the Faster R-CNN model. A comparison of the two models is shown in Fig. 6b. The mAP@0.5:0.95 of Faster R-CNN with data augmentation was 0.48. In terms of R^2^, the correlation of the test value with the real value, Faster R-CNN with FPN structure was 0.8676, 0.8403 and 0.812 in the categories of "open", "close", and “all”, respectively. Comparing these to the case without the FPN structure, the mAP@0.5:0.95 of the improved model increased by 0.002 (Fig. 7, Models 1 and 3), the R^2^ of the "close" class increased by 0.003, and the R^2^ of the "open" class and "all" class decreased slightly (Additional file 4: Table S4).

### Comparison of detection results with respect to data augmentation

The traditional Faster R-CNN model was constructed without data augmentation. To avoid the effect of sample imbalance, many kinds of data augmentation methods were added to the basic model, such as mixup and cutmix. The model was trained with and without data augmentation were trained and tested on the same dataset, and these detection results and correlations with the real numbers of manual labeling were compared. A comparison of the two models is shown in Fig. 6c. We found that the mAP@0.5:0.95 of Faster R-CNN with data augmentation was 0.494, which was 0.016 higher than that of Faster R-CNN without data augmentation (Fig. 7, Models 1 and 2). For the R^2^ ${R}^{2}$ of the correlation of the test value with the real value, Faster R-CNN with data augmentation was 0.8579, 0.8401 and 0.8235 in the categories of "open", "close", and “all”, respectively. The R^2^ in the category of "close" and “all” of Faster R-CNN with data augmentation were 0.0028 and 0.0035 higher than those of Faster R-CNN without augmentation. However, R^2^ in the "open" category of Faster R-CNN with data augmentation was 0.0133 lower than that of Faster R-CNN without data augmentation. Overall, the evaluation showed that the performance of Faster R-CNN with data augmentation is higher than that of Faster R-CNN without data augmentation (Additional file 5: Table S5).

### Comparison of detection results with respect to Multi-Scale

To test whether the multi-scale training can improve the detection accuracy of the quantity of dehiscent anthers, we added multi-scale on the basis of the traditional Faster R-CNN model. The specific content was obtained from the image pyramid at different scales and then the extracted features of the different scales for each layer of images, which was used to form the final feature map. Finally, the features of each scale were are individually predicted. A comparison of the two models is shown in Fig. 6d. The results showed that the mAP@0.5:0.95 of the model was improved by 0.003 after Multi-Scale training (Fig. 7, Models 4 and 2). However, R^2^ in the "open" and "close" categories fell by 0.0092 and 0.0007, respectively. R^2^ in the "all" category creased to 0.0086. Thus, Multi-Scale training has a certain effect on our research goal of cotton anther identification (Additional file 6: Table S6).

In this study, the change curves of each model in mAP@0.5:0.95 during the training process are shown in Fig. 7. The peak value of the traditional Faster-CNN mAP@0.5:0.95 curve was the lowest, while the peak value of the Faster R-CNN model with data augmentation, Multi-Scale training and FPN structure was the highest. The loss curve of each model during the training process is shown in Fig. 7. At the end of the training, the loss curve of the four models has tended to be stable.

### Screening of HT-tolerant cotton germplasms based on cotton anther phenotype data obtained using the integrated Faster R-CNN model

To select high temperature (HT) tolerant cotton germplasms, anther images of different cotton lines were obtained under normal temperature (NT) and HT. Then we counted the dehiscence status of anthers from 30 different cotton lines by manual observation and machine recognition. The statistical results are shown in Table 1. The manual observation results showed that the average dehiscence rates of cotton anthers treated with NT and HT were 84.35% and 35.46%, respectively. The results of machine recognition showed that the average dehiscent rates of cotton anthers treated with NT and HT were 83.81% and 35.08%, respectively. First, we believe that for the acquisition of the phenotypic data of the cotton anther dehiscence rate, the result of machine recognition has been extremely accurate, and the recognition speed is fast, which is not affected by artificial subjective factors, while saving manpower and material resources. There are obvious advantages compared with manual observation. Second, there is a great difference in the anther dehiscence rate of the same cotton variety between HT and NT conditions. The results show that HT greatly reduced the cotton anther dehiscence rate (Table 1), and then affected the pollination process, resulting in a reduction in cotton yield. Finally, by observing 30 cotton lines, we found that the anther dehiscence rate of S003 and S004 was still more than 85% under HT stress, which was significantly improved compared with that of the other lines (Table 1). In addition, we screened cotton lines with HT tolerance in large quantities through machine recognition, and obtained more than 35 HT tolerant cotton lines. These HT tolerant germplasms can be used in cotton HT tolerance breeding.

**Table 1:** Screening of HT tolerant cotton germplasms using ensembl Faster R-CNN model

	Manual count						Machine count					
	Normal temperature			High temperature			Normal temperature			High temperature		
Variety	Open	Close	Dehiscent rate (%)	Open	Close	Dehiscent rate (%)	Open	Close	Dehiscent rate (%)	Open	Close	Dehiscent rate (%)
S001	39.5 ± 2.02	5.75 ± 0.94	87.17 ± 2.3	34.75 ± 4.21	15 ± 4.76	70.41 ± 8.26	38.25 ± 2.39	5.5 ± 1.32	87.31 ± 2.97	35.25 ± 2.92	13.75 ± 3.9	72.47 ± 6.85
S002	26.25 ± 3.49	17.25 ± 1.37	78.6 ± 2.25	9.25 ± 2.14	17 ± 2.2	65.68 ± 3.41	26.75 ± 3.75	7 ± 1.29	79.35 ± 2.25	17.25 ± 1.31	8.5 ± 1.76	68.06 ± 3.13
S003	**42.25 ± 2.92**	**11.25 ± 2.01**	**78.84 ± 3.82**	**30 ± 6.17**	**4 ± 1.58**	**86.55 ± 5.03**	**40.25 ± 2.86**	**11 ± 1.87**	**78.54 ± 3.25**	**30.25 ± 5.76**	**5 ± 1.82**	**84.46 ± 5.56**
S004	**32.25 ± 2.28**	**3 ± 1.22**	**91.86 ± 3.02**	**27.25 ± 0.62**	**2 ± 0.91**	**93.27 ± 3.02**	**32.25 ± 2.28**	**2.25 ± 0.75**	**93.63 ± 1.92**	**27 ± 0.7**	**2.25 ± 0.75**	**92.4 ± 2.44**
S005	27.25 ± 3.9	9.75 ± 3.94	77.32 ± 7.54	29.25 ± 2.81	18.25 ± 5.12	36.19 ± 5.06	26.75 ± 4.42	10.25 ± 3.59	74.14 ± 6.48	17.5 ± 4.13	29.25 ± 2.95	36.12 ± 4.43
S006	28.75 ± 1.38	6 ± 1.08	83.97 ± 2.31	18.25 ± 1.37	13 ± 4.22	61.04 ± 9.65	28.25 ± 1.44	5.5 ± 0.65	83.86 ± 1.01	18.5 ± 0.86	12.75 ± 3.9	61.75 ± 8.76
S007	20.75 ± 3.59	5.75 ± 1.75	76.92 ± 8.33	16.25 ± 2.53	11.5 ± 1.19	42.3 ± 4.65	19.75 ± 2.68	6 ± 1.68	76.08 ± 7.37	10.75 ± 0.63	18 ± 2.27	37.94 ± 1.81
S008	17 ± 3.08	6 ± 0	72.69 ± 3.06	12.25 ± 0.75	18.5 ± 1.19	39.95 ± 2.81	18.25 ± 3.25	5.75 ± 0.25	74.95 ± 2.57	10.5 ± 0.28	19.5 ± 0.64	35.03 ± 0.95
S009	25 ± 2.85	4.5 ± 1.7	86.35 ± 3.53	13.5 ± 0.5	17 ± 2.94	45.62 ± 5.17	22.5 ± 2.72	5.5 ± 1.5	80.64 ± 4.45	13.25 ± 0.75	15.5 ± 2.25	46.97 ± 4.96
S010	24.25 ± 2.56	5.5 ± 1.44	82.31 ± 3.49	12.5 ± 1.44	21.75 ± 2.17	36.39 ± 1.82	23.75 ± 2.75	6 ± 1.58	80.87 ± 3.52	11 ± 0.57	21.5 ± 1.93	34.04 ± 1.27
S011	24.25 ± 4.49	4 ± 0.91	85.81 ± 1.41	8 ± 2.85	24.5 ± 6.73	19.77 ± 7.44	23.5 ± 4.34	3.25 ± 0.62	87.28 ± 1.94	8 ± 2.67	23.25 ± 6.14	20.43 ± 7.04
S012	28.25 ± 2.52	2.5 ± 0.5	91.51 ± 1.97	0 ± 0	29.25 ± 8.6	0 ± 0	26.5 ± 1.84	2.25 ± 0.62	91.9 ± 2.57	0.5 ± 0.5	28 ± 8.33	4.545 ± 4.54
S013	27.5 ± 6.06	3.75 ± 0.75	86.79 ± 3.53	0.25 ± 0.25	45 ± 5.11	0.5 ± 0.5	24.75 ± 5.45	4.25 ± 0.47	84 ± 3.15	0 ± 0	43.75 ± 3.19	0 ± 0
S014	29.75 ± 4.53	6.75 ± 2.09	81.08 ± 5.81	3.25 ± 3.25	37.25 ± 1.88	7.22 ± 7.22	28.5 ± 3.79	6.5 ± 2.21	81.08 ± 6.17	3.75 ± 3.42	38 ± 2.44	8.164 ± 7.44
S015	32.25 ± 1.03	3.75 ± 1.1	90.01 ± 2.61	17.25 ± 4.26	20.75 ± 3.01	44.65 ± 8.45	31.5 ± 0.64	3.75 ± 1.1	89.76 ± 2.71	16.75 ± 3.49	18.75 ± 1.43	45.98 ± 5.93
S016	22.75 ± 3.47	4 ± 1.58	86.06 ± 6.08	14.75 ± 0.63	7.25 ± 1.03	67.39 ± 3.2	22.25 ± 3.19	4.5 ± 1.19	83.23 ± 4.37	14 ± 0.58	7.5 ± 1.04	65.48 ± 3.34
S017	32.75 ± 2.39	8.25 ± 4.44	81.68 ± 9.2	12.5 ± 1.04	22.25 ± 4.39	37.12 ± 3.56	32 ± 2.27	8.5 ± 4.42	80.75 ± 9.66	12.5 ± 1.19	22.25 ± 3.68	36.64 ± 2.97
S018	31.5 ± 1.5	2.25 ± 0.47	93.42 ± 1.31	10.75 ± 0.95	21 ± 2.35	34.07 ± 1.61	30.5 ± 0.95	2.75 ± 0.47	91.75 ± 1.32	12.75 ± 0.75	21 ± 1.08	37.82 ± 2.19
S019	22.75 ± 1.03	3.25 ± 1.18	88.39 ± 4.22	14.25 ± 1.49	10 ± 1.63	58.91 ± 5.47	22 ± 0.91	3.5 ± 1.32	87.41 ± 4.48	13.75 ± 1.11	10.5 ± 1.85	57.4 ± 4.71
S020	29 ± 3.1	4.25 ± 1.18	86.98 ± 3.78	0.75 ± 0.48	29.5 ± 8.53	3.55 ± 2.8	26.25 ± 1.93	3.75 ± 1.03	87.58 ± 3.06	0.5 ± 0.5	30 ± 6.94	2.63 ± 2.63
S021	28 ± 0.41	3 ± 1.08	90.58 ± 3.16	10.75 ± 3.09	22.75 ± 6.34	32.5 ± 6.02	29.57 ± 0.85	3 ± 0.91	90.96 ± 2.6	9.75 ± 2.93	23 ± 5.4	29.34 ± 4.63
S022	29.5 ± 5.1	2 ± 0.91	93.95 ± 2.75	0.25 ± 0.25	45.25 ± 3.09	0.51 ± 0.51	28 ± 4.45	1.75 ± 1.03	94.65 ± 3.17	0 ± 0	43.75 ± 3.19	0 ± 0
S023	26.25 ± 2.09	2.5 ± 0.28	91.06 ± 1.51	0.5 ± 0.28	43.25 ± 6.42	1.045 ± 0.6	25.25 ± 1.37	2.5 ± 0.28	90.91 ± 1.19	0.5 ± 0.28	43.5 ± 6.33	0.994 ± 0.57
S024	30 ± 4.65	6.75 ± 1.7	80.44 ± 5.82	0.75 ± 0.48	37.75 ± 2.66	2.07 ± 1.43	28.5 ± 3.79	6.5 ± 2.21	81.08 ± 6.17	0.75 ± 0.75	38.5 ± 3.88	2.34 ± 2.34
S025	18.75 ± 3.32	7 ± 0.7	71.34 ± 5.16	13.5 ± 0.65	21.75 ± 3.33	39.36 ± 4.57	19.25 ± 3.17	8.5 ± 1.19	68.31 ± 5.9	14.5 ± 0.29	22.75 ± 3.17	39.82 ± 3.47
S026	32.5 ± 1.04	3.75 ± 1.1	89.99 ± 2.76	3 ± 1.68	41.75 ± 7.9	9.35 ± 6.76	31.5 ± 0.64	3.75 ± 1.1	89.76 ± 2.71	3 ± 2.34	42.25 ± 8.73	9.669 ± 8.28
S027	23 ± 3.82	4.75 ± 1.37	83.29 ± 3.76	13.5 ± 1.19	7.25 ± 0.75	65.03 ± 2.37	22.25 ± 3.19	4.5 ± 1.19	83.23 ± 4.37	14 ± 0.58	7.5 ± 1.04	65.48 ± 3.44
S028	31.25 ± 2.89	4.25 ± 1.49	87.53 ± 4.6	5.25 ± 1.93	20.5 ± 3.96	19.19 ± 4.81	31 ± 1.87	4.75 ± 1.18	86.58 ± 3.46	5.25 ± 2.75	21.25 ± 4.47	14.49 ± 7.04
S029	9.25 ± 3.07	2.75 ± 0.25	72.05 ± 6.26	0.5 ± 0.5	10 ± 0.82	3.57 ± 3.57	8.5 ± 3.18	2.5 ± 0.29	71.18 ± 6.45	0.5 ± 0.29	10 ± 0.91	4.42 ± 2.6
S030	21.75 ± 2.8	4.25 ± 0.25	83.15 ± 1.67	11.25 ± 0.25	18.25 ± 0.48	38.15 ± 0.57	20.75 ± 1.93	4 ± 0.4	83.79 ± 1.07	11.25 ± 0.85	17 ± 1.35	39.87 ± 0.67
Average	27.2 ± 0.36	4.95 ± 0.55	84.35 ± 1.23	11.21 ± 0.77	22.03 ± 0.96	35.46 ± 1.61	26.25 ± 0.31	4.98 ± 0.5	83.81 ± 1.17	11.11 ± 0.68	21.97 ± 0.81	35.08 ± 1.76

Open: the average number of dehiscent anthers from different flowers, n > 4

Close: the average number of indehiscent anthers from different flowers, n > 4

Dehiscent rate: the number of dehiscent anthers of each flower/(the number of dehiscent anthers of each flower + the number of indehiscent anthers of each flower), n > 4

Bold values indicate the anther dehiscence rate of S003 and S004 was still more than 85% under HT stress, which was significantly improved compared with that of the other lines

## Discussion

Through analysis, we found that the mAP@0.5:0.95 value of the model increased significantly after adding data augmentation, FPN structure and Multi-Scale, but the change of R2 was not significantly positively correlated with mAP@0.5:0.95. To obtain the most accurate data in the application, four models were trained, as shown in Fig. 7 and tested on the same batch of test sets. The recognition results obtained were integrated by the following formulae:

$$ {\text{result}}_{{{\text{open}}}} = \frac{{\sum\limits_{{{\text{i}} = 1}}^{{4}} {\mathop {{\text{model}}}\nolimits_{{\text{i}}}^{{{\text{open}}}} } }}{4} $$

$$ {\text{result}}_{{{\text{close}}}} = \frac{{\sum\limits_{{{\text{i}} = 1}}^{{4}} {\mathop {{\text{model}}}\nolimits_{{\text{i}}}^{{{\text{close}}}} } }}{4} $$

Among those, i represents the number of the model in Fig. 7. Model_i_^open^ represents the number of dehiscent cotton anthers identified by model_i_ in the verification set. Model_i_^close^ represents the number of indehiscent cotton anthers identified by model_i_ in the verification set.

After the comparison with the real value, it is found that when the model is integrated, the detection result after ensemble effectively compensates for the error, and the correlation between the detection result and the real value increases. After the ensemble of the four models, R^2^ of “open” reaches to 0.8765, R^2^ of “close” reaches to 0.8539, and R^2^ of “all” reaches to 0.8481, higher than the prediction result of either model alone. Therefore, when accurate data are needed, we can choose to integrate the detection results of the four models so that the detection data are the most reliable. Of course, directly using the Faster R-CNN model with FPN structure, data augmentation and multi-scale has higher robustness and higher accuracy.

It is well known that anthers are the male organs of plants, and anther abortion will directly lead to male sterility and reduce yield. Our previous studies preliminarily concluded that HT stress can reduce cotton yield by inhibiting cotton male fertility. HT mainly decreased pollen viability, anther growth number, and the percentage of dehiscent anthers, causing the decreases in male fertility in cotton [15, 16]. Furthermore, with the development of sequencing technology, a large amount of cotton germplasm resequencing data and transcriptome variation data have been obtained [14, 27]. However, no genes that enhance HT tolerance in male reproductive organs have been cloned. The main reason is that it is difficult to obtain phenotypes of reproductive organs. Thus, in this study, we built and trained an augmented Faster R-CNN rapid identification system of cotton anther phenotype, which can quickly investigate the anther phenotype and can be used to locate of the genes affecting cotton anther dehiscence under HT by combining the genome-wide association study and whole transcriptome association study. This will effectively promote cotton HT tolerance breeding and ensure safe cotton production despite the trend of global warming.

## Conclusions and future directions

### Conclusions

1. In this paper, a high-throughput cotton anther phenotype recognition system is proposed based on deep learning. It takes 1 min or even longer to manually count the anther dehiscence state of a cotton, while it only takes 1 s to detect the state from each image using a deep learning model. This is the first time that a deep learning technique has been applied to the detection of cotton anther phenotypes. The computer model is trained by deep learning instead of manually completing the statistics of cotton anther phenotype. The problems related to time-consumption and low accuracy of manual counting of anther phenotype data in the past are solved, helping researchers to quickly study the anther phenotypes of cotton. Then the response genes of cotton anthers to stress can be located, and used for breeding and improvement.
2. A lightweight cotton anther dehiscence detection model based on YOLOv5 is proposed, which can be easily implanted into embedded devices or mobile devices.
3. Through the reported changes in the accuracy and correlation of Faster R-CNN after the improvement of the data augmentation method, the feasibility and superiority of the improved method are verified.
4. After the ensemble of the four models, R^2^ of “open” reaches to 0.8765, R^2^ of “close” reaches to 0.8539, R^2^ of “all” reaches to 0.8481, which are higher than the prediction result of either model alone, and can completely replace the manual counting method. This study provides new technical support for cotton reproductive development and HT tolerance breeding.
5. In the past, the high-throughput detection of cotton phenotypes was often aimed at the field composed of whole cotton or multiple cotton plants, and the detection tasks included cotton agricultural damage detection [7, 8] and cotton yield prediction [9]. Our research is different from the past: we focus on the small goal of cotton anthers. It takes 1 min or even longer to manually count the anther dehiscence state of cotton flower, but it only takes 1 s to detect each image using a deep learning model. This is the first study to achieve high-throughput detection of the cotton anther dehiscence state.

### Future directions

In this study, YOLOv5 and Faster R-CNN are applied to identify the dehiscence status of cotton anthers and achieved fast and accurate identification. However, there are still some areas where there is room for improvement:

1. We examined the dehiscence of cotton anthers, but other phenotypes such as the growth position of anthers and the distance between anthers and stigmas are also important for cotton fertility under HT. Other phenotypic characteristics of cotton anthers can be collected by using a comprehensive platform that integrates multiple data points to analyze cotton reproductive development.
2. The cotton anther dehiscence recognition model trained in this study should be further developed and applied to mobile devices to facilitate cotton reproductive development. Researchers should use the model to obtain anther dehiscence data quickly and accurately at any time during agricultural activities.
3. In this study, the experience of deep learning model training for cotton anther dehiscence can be applied to other plant anther state detection. It is one of the directions to further enrich the construction of multi-crop anther state recognition model based on deep learning.
4. To provide data support for the study of cotton reproductive development, in addition to cotton anthers, the detection of other phenotypic traits of cotton should also be considered. For example, reading the number and growth position of cotton peach, the quantitative relationship between fruit bud and leaf bud and other traits, we can also use similar research methods, and build a high-throughput detection model to help researchers quickly and accurately obtain phenotypic data.

## Supplementary Information

**Additional file 1: Table S1.** Dataset.

**Additional file 2: Table S2.** Experimental configuration.

**Additional file 3: Table S3.** Comparison of YOLOv5 and Faster R-CNN.

**Additional file 4: Table S4.** Comparison of FPN.

**Additional file 5: Table S5.** Comparison of data augmentation.

**Additional file 6: Table S6.** Comparison of Multi-Scale.

## Data Availability

The data set offers the dehiscence of cotton anthers under high temperature stress, so data is temporarily unavailable. As the experiment progresses, our data will gradually improve the available state.

## Abbreviations

AUPRCs: Areas under precision/recall curves
ERM: Empirical risk minimization
FPN: Feature pyramid networks
HT: High temperature
lncRNAs: Long noncoding RNAs
MAD: Mean absolute deviation
NLP: Natural language processing
NT: Normal temperature (NT)
RPN: Region proposal network
SSD: Single shot multibox detector
SVM: Support vector machine
